# The Mediating Role of School Refusal in the Relationship Between Students’ Perceived School Atmosphere and Underachievement

**DOI:** 10.3390/ejihpe15010001

**Published:** 2024-12-31

**Authors:** Luana Sorrenti, Concettina Caparello, Carmelo Francesco Meduri, Pina Filippello

**Affiliations:** 1 Department of Clinical and Experimental Medicine, University of Messina, 98100 Messina, Italy; gfilippello@unime.it; 2 Department of Health Sciences, University Magna Graecia of Catanzaro, 88100 Catanzaro, Italy; concettina.caparello@unicz.it (C.C.); carmelofrancesco.meduri@unicz.it (C.F.M.)

**Keywords:** school refusal, school atmosphere, underachievement, school engagement

## Abstract

Studies have shown that the school atmosphere perceived by students can play a key role in promoting their well-being and success in school. No study to date has analyzed whether the students’ perceived school atmosphere might contribute to school refusal (SR), which in turn might reduce students’ engagement and promote underachievement. A cross-sectional study was conducted with 528 Italian high school students (M_*age*_ = 16.08, *SD* = 1.38; 50.8% males, 47% females, and 2.3% not declared), with the aim of assessing the role of the mediation of SR (Anxious Anticipation, Difficult Transition, Interpersonal Discomfort, and School Avoidance) in the association between students’ perceived school atmosphere (Student Relations, Student–Teacher Relations, Educational Climate, Sense of Belonging, and Interpersonal Justice) and school engagement and underachievement. Data were collected using validated instruments, including the SChool REfusal EvaluatioN for school refusal, the Multidimensional School Climate Questionnaire for school atmosphere, and the Utrecht Work Engagement Scale for school engagement. To evaluate the association between variables, we performed structural equation modeling with latent variables. Mediation analysis indicated that Difficult Transition fully mediates the association between Sense of Belonging and school engagement (β = 0.20, *p* ≤ 0.05). This study extends the knowledge of school refusal behavior.

## 1. Introduction

### 1.1. School Refusal and Underachievement

School refusal is characterized by the youth’s experience of strong negative emotions when attending school or faced with the prospect of going to school. In the case of school refusal, therefore, non-attendance is linked to the experience of strong negative emotions when attending school. These negative emotions are often the source of absenteeism at school, which occurs through, inter alia, being tardy to school frequently, visiting the school infirmary and/or the school office frequently, and calling their parents to leave school and return home (Gallé-Tessonneau & Heyne, 2020). According to Gallé-Tessonneau and Gana (2019), adolescents may refuse to attend school for four main reasons: Anticipatory Anxiety, Difficult Transition, Interpersonal Discomfort, and School Avoidance. School refusal due to Anticipatory Anxiety is related to anxiety about attending school and fear of facing the demands of school. School refusal due to Difficult Transition refers to a student’s difficulty in leaving home and parents to go to school and, thus, difficulty in coping with the transition and separation. School refusal due to Interpersonal Discomfort is related to difficulties in interpersonal relationships in the school context. School refusal due to School Avoidance refers to an adolescent’s difficulties in managing and regulating emotional and psychological distress that is expressed through somatic manifestations. According to Havik et al. (2015), the reasons behind school refusal encompass individual-related, family-related, and school-related factors. Among school-related factors, school atmosphere is an important factor in explaining school attendance problems (Hamlin, 2021; Van Eck et al., 2017). However, while the relationship between specific dimensions of school atmosphere with school attendance problems has been investigated (Kotok et al., 2016) few existing studies have integrated multiple perspectives on different aspects and dimensions of school atmosphere to examine its association with school refusal (Molinari & Grazia, 2023) and underachievement (Chere & Hlalele, 2014; Kapri, 2017), the latter being generally understood as “failure to meet the academic requirements of the school setting” (Connor, 2002, p. 133). Underachievement can lead to lower levels of self-esteem, task salience (Durán-Narucki, 2008), and performance patterns (Snyder et al., 2021). Conversely, good academic achievement is crucial for success in higher education and increases the likelihood of students succeeding in school and entering the workforce (Hayek et al., 2022; Rai & Khanal, 2017).

### 1.2. School Refusal and School Atmosphere

School atmosphere refers to students’ perceptions of the intangible and abstract characteristics of the perceived relational and educational environment of the school, which students develop in their daily lives in the classroom, through repeated interactions with other students and with teachers and through involvement and participation in classroom practices (Molinari & Grazia, 2023; Hendron & Kearney, 2016). Through all these actions, they construct their experience as members of the school community and develop interpretations and perceptions that converge in their relational (Sense of Belonging, Interpersonal Justice, and relations with the teachers and other students) and educational (Educational Climate) views of the school atmosphere (Molinari & Grazia, 2023; Rudasill et al., 2018). A better understanding of the relationship between school refusal, underachievement, and school atmosphere can be achieved by identifying typical patterns in students’ perceptions of their school environment (Kotok et al., 2016; Vaillancourt et al., 2013). Pupils’ Sense of Belonging refers to their perception of feeling connected to the school. Schools characterized by a high sense of school belonging and good school disciplinary climate were less likely to be plagued by students skipping classes (Keppens & Spruyt, 2019). Indeed, a sense of connectedness at school has the potential to satisfy developmental needs for social connectedness and relationships with others, which can increase motivation for school attendance (Gershenson, 2016; Simons et al., 2010). Conversely, feeling insecure, unaccepted, and lacking a Sense of Belonging among peers at school contribute to student alienation, failure, and disaffection from school, leading to school absenteeism (Baskerville, 2020; Daily et al., 2020). Pupils’ perception of Interpersonal Justice in their educational environment refers to their perception of being “treated fairly, with dignity and respect in interpersonal interactions” (Chory-Assad, 2002, p. 61). Experiences of being treated fairly and respectfully in the school context, particularly during adolescence, contribute to perceptions of being a valued member of the group and promote students’ Sense of Belonging, motivation, and engagement (Gubbels et al., 2019). Furthermore, students who feel justly treated by their teachers are more likely to attend school and less likely to refuse school (Grazia et al., 2024). Conversely, students who frequently refuse to go to school feel that they are treated more unfairly by their teachers (Donat et al., 2018). Another component of the school atmosphere is the quality of interpersonal relationships that are created between pupils and between one pupil and a teacher. Teacher–student relationships refer to the educational and emotional support students receive from their teachers (O’Brennan & Bradshaw, 2013; Wang & Degol, 2016). Indeed, positive student–teacher relationships can help students strive for higher educational achievement (Benbenishty et al., 2016), regulate their emotions, and build social skills (Konold et al., 2018; Osher et al., 2020), and they provide a buffer against risk factors for absenteeism (Adams et al., 2016). Therefore, relationships characterized by trust, respect, and fairness between teachers and students can generate positive social norms and emotional support systems that help reduce School Avoidance (Hamlin, 2021) and school refusal (Havik et al., 2015). These interpersonal relationships are an important part of the school engagement processes that make students feel connected and remain in school (Contreras et al., 2022). However, while some studies have found that positive student–teacher relationships reduce the risk of school refusal (Ingul et al., 2019; Khursheed et al., 2021), Lessard et al. (2008) found no relationship between teacher–student relationships and school abandonment. Much less research has been conducted on the quality of interpersonal relationships between students and absenteeism rates. Orpinas and Raczynski (2016) showed that being the victim of gossip or lies, being deliberately excluded, or being forced to do things to be liked by peers increases the likelihood of school absenteeism. This makes it difficult for students to stay in a class where they feel unwanted and disliked, and therefore, they are more likely to have high rates of absenteeism (Gubbels et al., 2019), which can often be responsible for unsatisfactory academic achievements (Chere & Hlalele, 2014). However, in agreement with Havik et al. (2015), there is a need for further research into the role of peer relationships in school refusal. Another dimension of the school atmosphere is the Educational Climate, which refers to students’ perception of the learning environment, including teaching activities, teacher expectations, and orderliness (Karlberg et al., 2022). Previous research (Karlberg et al., 2022; Fortin et al., 2006) has shown that a lack of order, structure, or organization in the classroom, curricula poorly tailored to individual student needs, and inflexible disciplinary strategies can be risk factors for non-attendance in general and for school refusal. Moreover, if the school environment is inadequate or impoverished, effective learning cannot take place, affecting the way students view school and learning and leading to unsatisfactory academic achievements (Chere & Hlalele, 2014). Conversely, an Educational Climate in which classroom activities are well-structured may promote students’ sense of predictability or perceived control over the school environment (Havik et al., 2015). However, most studies have focused on examining the components of the school atmosphere either together or individually, and few studies have examined the relationship between absenteeism and educational atmosphere specifically, even though patterns of school atmosphere are an important part of the process of school engagement (Büchele, 2021).

### 1.3. School Refusal and School Engagement

School engagement (SE) is defined as the energy in action that a student uses at the behavioral (i.e., time spent on tasks), emotional (i.e., students’ feelings and connections to school), and cognitive (i.e., self-regulation and learning strategies) levels in the educational context (Fredricks & McColskey, 2012). Indeed, students who feel engaged in school tend to report better educational (better grades and acquisition of knowledge and skills) and social outcomes (Li & Lerner, 2011). Conversely, repeated occurrences of tardiness or leaving school early may indicate disengagement (Wang et al., 2019). Therefore, an educational environment that is free from worries fosters a Sense of Belonging and is characterized by good interpersonal relationships with teachers and other students would encourage students to be more engaged in learning, to attend school (Bauer et al., 2018; Freeman et al., 2016), and to achieve better academic results (Chere & Hlalele, 2014). In addition, the perception of being treated fairly by teachers promotes student engagement, probably because the perception of being treated fairly results in an atmosphere of trust and mutual respect, which promotes student engagement (Molinari & Grazia, 2023). Conversely, students who perceive that the school environment does not meet their educational needs, that teachers exercise excessive control and discipline over them, that the school atmosphere is characterized by high levels of distrust and disrespect between students and teachers, and that students do not care about each other, as well as those who report receiving negative comments and other criticisms from their teachers, are more likely to experience feelings of inadequacy and anxiety about school and to feel less engaged in school (Karlberg et al., 2022; Rizzotto & França, 2022).

### 1.4. Relationship Between School Atmosphere, School Refusal, and Success

Research has highlighted the role of school-related and personal factors in shaping school attendance issues, including behaviors such as school refusal. For example, school variables, such as the overall school atmosphere (Molinari & Grazia, 2023; Rudasill et al., 2018), and personal factors, like school performance and engagement (Chere & Hlalele, 2014; Kapri, 2017; Bauer et al., 2018), have been shown to play significant roles in attendance. A positive school atmosphere, marked by supportive interpersonal relationships, perceived fairness, and a well-structured educational environment, has been found to enhance students’ Sense of Belonging and engagement (Bauer et al., 2018). This supportive environment not only motivates students to attend school regularly but also promotes active participation in school activities, contributing to improved academic performance (Hamlin, 2021; Bacon & Kearney, 2020). Conversely, a school atmosphere perceived as negative or confrontational diminishes students’ participation and engagement. Such environments may heighten feelings of anxiety and discomfort associated with school, increasing the likelihood of school refusal behaviors (Havik et al., 2015; Hamlin, 2021). In this context, school refusal emerges as a critical mediator in the relationship between the school atmosphere and academic outcomes, such as underachievement (Durán-Narucki, 2008). By addressing these dynamics, educators and policymakers can better understand the intricate interplay of environmental and individual factors influencing school attendance.

## 2. The Present Study

Fostering a positive school atmosphere can be an effective way of promoting well-being and student attendance (Rizzotto & França, 2022; Virtanen et al., 2023). Indeed, quality interpersonal relationships, Sense of Belonging, perceptions of school justice, and an Educational Climate that supports and encourages students in their autonomy can protect pupils from factors that can lead to absenteeism and thus increase their motivation to attend school (Hamlin, 2021). Therefore, students’ perceptions of a good school atmosphere, increased engagement, and success at school were found to be correlated with a lower risk of absenteeism (Contreras et al., 2022; Bacon & Kearney, 2020; Glaesser et al., 2024). In contrast, underachievement and low levels of school safety, personal relationships, and school engagement were associated with a higher risk of absenteeism (Havik et al., 2015; Karlberg et al., 2022; Buzzai et al., 2021a).

Moreover, most studies emphasize the positive effects of a good school atmosphere on pupils’ attendance, involvement, and commitment (Wang & Degol, 2016). For example, absenteeism problems are often linked to conflictual relationships between teachers and pupils (Contreras et al., 2022), negative peer dynamics (Havik et al., 2015), and a lack of belonging to the school (Ingul et al., 2019). Conversely, higher pupil attendance appears to be closely associated with a positive, welcoming, and motivating school environment (Hamlin, 2021; Bacon & Kearney, 2020), which fosters a greater Sense of Belonging and active participation (Virtanen et al., 2023). While previous studies have often examined the relationships between these variables individually, this study provides a more integrated analysis by exploring their interplay within the context of school refusal. Indeed, to the best of our knowledge, there are no studies that have analyzed whether students’ perceived school atmosphere might contribute to school refusal and, in turn, reduce/promote students’ school engagement and underachievement. The literature still lacks a comprehensive model that explains the relationships between students’ perceived school atmosphere, school refusal behavior, students’ school engagement, and underachievement. Therefore, the purpose of this study was to examine the mediating role of school refusal (Anxious Anticipation, Difficult Transition, Interpersonal Discomfort, and School Avoidance) in the association between students’ perceived school atmosphere (Student Relations, Student–Teacher Relations, Educational Climate, Sense of Belonging, and Interpersonal Justice) and school engagement and underachievement. Based on previous research and in accordance with Molinari and Grazia’s (2023) model of the school atmosphere and Gallé-Tessonneau and Gana’s (2019) model of school refusal, we expected that one or more dimensions of students’ perceived school atmosphere would have a direct relationship with one or more school refusal behaviors and, in turn, an indirect relationship with students’ school engagement and underachievement. We hypothesize that school atmosphere (Student Relations, Student–Teacher Relations, Sense of Belonging, and Interpersonal Justice) can reduce school refusal and, consequently, increase school engagement and reduce underachievement. Moreover, we hypothesize that a rigid Educational Climate that does not meet pupils’ educational needs can promote school refusal and, consequently, reduce school engagement and promote underachievement. According to our knowledge, no study has examined the different dimensions of school atmosphere in relation to school refusal separately and simultaneously, and there is also a lack of studies that have examined the relationship between Educational Climate and school refusal in depth. Furthermore, to explore the role of school climate in depth, our model analyzed five aspects of school climate separately.

## 3. Materials and Methods

### 3.1. Participants

The present study involved 528 high school students aged between 14 and 20 years (M_*age*_ = 16.08; *SD* = 1.38), with no missing data reported. The sample consists of 248 male students (50.8%), 268 female students (47%), and 12 students (2.3%) who did not declare their sex. Participants were recruited from public high schools in Southern Italy, specifically from the city of Siracusa and its surrounding province (Sicily). Most students (402; 76%) lived in the city, while 24% (127 students) resided in the broader province.

In Italy, high school lasts five years and is typically attended by students aged 14 to 19. It is divided into two main stages: the biennium (first two years, ages 14–15) and the triennium (final three years, ages 16–19). Regarding the distribution of students across grade levels, 67 students (1.7%) were enrolled in the first year and 49 students (9.3%) were enrolled in the second year, both of which constitute the biennium. In the triennium, 165 students (31.2%) were in the third year, 119 students (22.7%) were in the fourth year, and 128 students (24.2%) were in the fifth year. To evaluate the distribution of the sample with respect to gender and age, the latter being categorized as the biennium and the triennium, a Chi-square analysis was performed to assess sample homogeneity. The results showed no statistically significant differences (χ^2^ = 0.07, df = 2, *p* > 0.05), indicating that gender was evenly distributed across the different age groups. In terms of school performance, the students reported an average grade of 8.07, (*SD* = 0.89), reflecting the overall academic achievement across the sample. Grades ranged from a minimum of 5 to a maximum of 10, consistent with the Italian grading system, where scores from 1 to 5 are insufficient and 6 is the minimum passing grade. A grade of 6 indicates that a student has met the essential requirements, while grades from 7 to 8 reflect good to very good performance. Higher scores, such as 9 and 10, are considered excellent, with a grade of 9 demonstrating a strong grasp of the subject and a grade of 10 representing outstanding achievement. Regarding the number of absences, students were asked to indicate the total number of absences (M = 20.83; *SD* = 10.98) and the number of unexcused absences (M = 1.75; *SD* = 7.35). Regarding students’ socioeconomic status (SES), 28% of the students belonged to a low SES (one or both parents held a lower secondary education diploma), 45.5% belonged to a medium SES (one or both parents held a high school diploma), and 26.5% belonged to a high SES (one or both parents held a university degree). Furthermore, 99% of the students had an Italian nationality (524 students), and all participants spoke Italian. Students with intellectual disabilities or special educational needs did not participate in the research.

### 3.2. Instruments

A demographic questionnaire was used to collect the participants’ basic demographic information, including their age, gender, nationality, educational level, and socioeconomic status (SES). Underachievement scores were used to measure students’ lack of success during their studies, such as failing the course and having to repeat a school year. Italian students can experience two different negative situations related to school failure. The first is characterized by failure. In this situation, there are students with different levels of insufficiency in many subjects (such as four or five subjects) and they must repeat the school year. The second situation is characterized by deficiencies in a few subjects (e.g., two or three subjects) and the students can reduce their knowledge gaps by continuing their studies. A questionnaire was used to collect basic information about the participants, including school grades, failure, and absenteeism rates. The School Atmosphere subscale of the Italian version of the Multidimensional School Climate Questionnaire (MSCQ) (Grazia & Molinari, 2021) was used to measure the school atmosphere. The subscale consists of 22 items on a 6-point Likert scale (from 1 = “strongly disagree” to 6 = “strongly agree”), grouped into five factors: Student Relations (SR; e.g., “Students help each other”), Student–Teacher Relations (STR; e.g., “Students feel close to most of their teachers and they trust them”), Educational Climate (EC; e.g., “At my school, you can feel that students’ success is the priority for teachers”), Sense of Belonging (SB; e.g., “I love my school”), and Interpersonal Justice (IJ., e.g., “Students are treated with justice”). Every item started with “In this school…”. The MSCQ demonstrated acceptable reliability and construct validity in previous studies (Molinari & Grazia, 2023). In this study, the scale had good internal reliabilities (α = 0.85).

The SChool REfusal EvaluatioN (SCREEN) (Gallé-Tessonneau & Gana, 2019) is a self-administered 18-item questionnaire designed to assess school refusal in adolescents. Among the various instruments available in the literature to examine school refusal, the SCREEN stands out for its ability to analyze four relevant components of school refusal in a targeted and thorough manner (Gonzálvez et al., 2021): Anticipatory Anxiety (AA; e.g., “I feel like I have a mental block when it comes to going to school, as is if I won’t be able to”), Transition Difficulties (DT; e.g., “I tell my parents that I don’t want to go to school and I want to stay at home”), Interpersonal Discomfort (ID, e.g., “I’m afraid of what others in my class think of me”), and School Avoidance (SA, e.g., “I’m absent more often this year than last year”). For each item, students can respond on a 5-point Likert scale (“Doesn’t apply to me at all” = 1, “Applies to me a little” = 2, “Applies to me somewhat” = 3, “Applies to me a lot” = 4, and “Applies to me completely = 5”). The SCREEN was also selected for its psychometric validity, having demonstrated acceptable reliability and construct validity in previous studies (Gallé-Tessonneau & Heyne, 2020; Boudouda et al., 2024). In this study, the questionnaire had good internal reliabilities (α = 0.86).

The Italian version of the Utrecht Work Engagement Scale (UWES-9) (Balducci et al., 2010) was used to measure school engagement in the academic setting. The UWES-9 consists of 9 self-reported items (e.g., “I am enthusiastic about my study”). The scale includes three subscales: Vigor, Dedication, and Absorption. In this study, the composite score was used. For each item, participants responded on a 7-point Likert scale ranging from 0 (“never”) to 6 (“Always/every day”). The UWES-9 demonstrated acceptable reliability and construct validity in previous studies and different countries (Schaufeli & Bakker, 2004; Buzzai et al., 2021b). In this study, the scale had good internal reliabilities (α = 0.92).

### 3.3. Procedure

This study was performed following the recommendations of the Ethical Code of the Italian Association of Psychology (AIP). Once approved, the principals of the selected secondary schools, located in the south of Italy, were contacted to obtain the necessary permission for students to participate in this study. Students, teaching staff, and families were given all the relevant information to encourage everyone’s participation in the research. The students’ families signed the informed consent forms after understanding the aims and objectives of this study. They were fully aware that their cooperation in the research was based on the guarantee of anonymity and the possibility for their children to withdraw from this study at any time. All subjects agreed to participate in the research project and were granted their written informed consent in accordance with the Declaration of Helsinki (2013). The protocol was approved by the University of Alicante [UA-2023-06-29-4]. Only participants who returned signed informed consent forms were allowed to participate in this study. After consent was obtained, students completed the questionnaires in a single session during school hours. Before administration began, they were given instructions about the purpose of this study and the importance of their voluntary participation. Each completion session was supervised, providing a controlled environment and an opportunity to clarify any doubts the students might have. Participants’ privacy and anonymity were guaranteed. Students needed 15 to 20 min to complete the questionnaires.

### 3.4. Data Analysis

Jamovi software 2.3.28 was used to calculate descriptive statistics and Cronbach’s alpha. The lavaan package in RStudio was used to carry out structural equation modeling (SEM) of the latent variables (Rosseel, 2012). The SEM approach reduces the probability of type-I errors and has been demonstrated to be superior to traditional univariate and multivariate approaches (Iacobucci et al., 2007; Kline, 2015). Moreover, this approach provides the opportunity to specify latent variables rather than measured variables because measured variables are assumed to be measured without error (Coffman & MacCallum, 2005). SEM with latent variables treats the constructs measured using the questionnaire as the latent variables, and multiple indicators are required for all constructs evaluated. Each latent construct’s parcel consisted of the aggregated mean on a common scale of group items from the questionnaire items to which the participants responded. Parcels (groups) of items for all the constructs examined in this research were used as indicators (Little et al., 2002). The parceling procedure improves commonality across indicators, reduces random error, increases modeling efficiency, and yields normalized distributions rather than individual items and total scale scores (Coffman & MacCallum, 2005; Bagozzi & Edwards, 1998; Hau & Marsh, 2004; Kishton & Widaman, 1994; MacCallum et al., 1999; Matsunaga, 2008). Indexes of model fit are usually more acceptable when parcels, rather than items, are modeled because of the psychometric and estimative advantages of parcels. In this study, we used the confidence intervals of direct and indirect effects with 5000 bootstrap replication samples. In accordance with Wu and Jia (2013), Preacher and Hayes (2008), and Shrout and Bolger (2002), a 95% bias-corrected confidence interval (CI) was applied. Several indexes of fit were examined: the Chi-square value, χ^2^/df, the comparative fit index, and the root mean square error of approximation with its 90% confidence interval (CI). The cutoff for a good model fit was CFI values > 0.90 and RMSEA values < 0.08 (Kline, 2015). Gender and age were also included in the model as control variables.

## 4. Results

### 4.1. Correlation

The correlational analysis highlights key relationships between variables related to perceived school climate, school engagement, school refusal, and academic underachievement (Table 1). Negative correlations were found between school engagement and several dimensions of school refusal, particularly Anticipatory Anxiety and Difficult Transition. This suggests that higher engagement is associated with fewer challenges in these areas. Similarly, school engagement is negatively correlated with perceptions of several dimensions of school atmosphere, including relationships among students, relationships between students and teachers, and overall Sense of Belonging, suggesting that lower perceptions of these positive aspects of school climate may be associated with lower engagement. Conversely, perceptions of a positive school climate, particularly in terms of the quality of interpersonal relationships, are associated with higher levels of engagement and lower levels of school disengagement. Finally, underachievement is positively correlated with the dimensions of school refusal, suggesting that difficulties in attending school may be related to lower school performance. The correlation analysis showed a strong correlation between the variables studied. These preliminary data have made it possible to construct an appropriate model that considers the relationship between school refusal, school atmosphere, underachievement, and school engagement.

### 4.2. Mediation

Structural equation modeling (SEM) was employed with latent variables to investigate the mediating role of school refusal (Anxious Anticipation, Difficult Transition, Interpersonal Discomfort, and School Avoidance) in the association between students’ perceived school atmosphere (Student Relations, Student–Teacher Relations, Educational Climate, Sense of Belonging, and Interpersonal Justice) and school engagement and underachievement. SEM was chosen for this analysis because it allows for the simultaneous examination of multiple relationships between variables, making it particularly suitable for testing mediation models involving complex constructs. The mediating role of the four school refusal dimensions, as outlined by Gallé-Tessonneau and Gana (2019), was of particular interest in this study. This approach enabled us to explore how the school atmosphere is indirectly related to students’ engagement and academic outcomes. By examining these indirect pathways, we sought to uncover the specific role of school refusal behaviors in shaping the broader impact of the school atmosphere on student performance and participation. The estimation of the model demonstrated a good overall fit to the data, indicating that the proposed structure adequately captured the relationships among the variables. The fit indices were as follows: χ^2^ (270) = 655.239, *p* = 0.000, CFI = 0.95, SRMR = 0.04, and RMSEA (90% CI) = 0.052 (0.047, 0.057).

**Figure 1:**
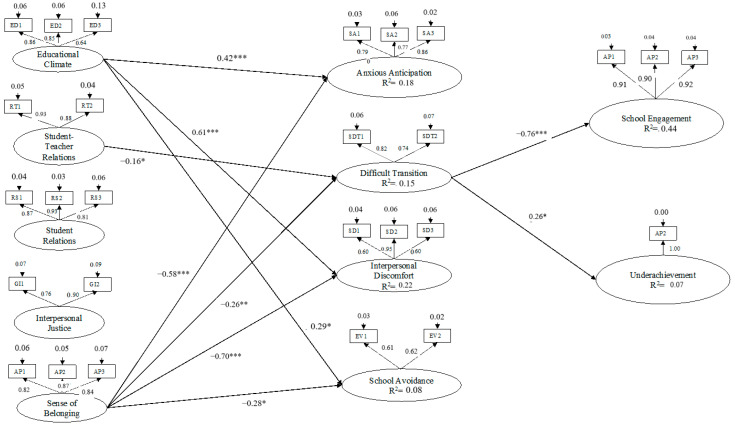
Full Mediation Model. Note: *** *p* ≤ 0.001, ** *p* ≤ 0.01, and * *p* ≤ 0.05. The coefficients shown are standardized direct path coefficients. The insignificant paths have not been inserted. Coefficients’ correlation: Educational Climate ↔ Student–Teacher Relations: 0.65 ***; Educational Climate ↔ Student Relations: 0.64 ***; Educational Climate ↔ Interpersonal Justice: 0.68 ***; Educational Climate ↔ Sense of Belonging: 0.79 ***; Student–Teacher Relations ↔ Student Relations: 0.63 ***; Student–Teacher Relations ↔ Interpersonal Justice: 0.65 ***; Student–Teacher Relations ↔ Sense of Belonging: 0.62 ***; Student Relations ↔ Interpersonal Justice: 0.53 ***; Student Relations ↔ Sense of Belonging: 0.63 ***; Interpersonal Justice ↔ Sense of Belonging: 0.64 ***; Anxious Anticipation ↔ Difficult Transition: 0.67 ***; Anxious Anticipation ↔ Interpersonal Discomfort: 0.52 ***; Anxious Anticipation ↔ School Avoidance: 0.68 ***; Difficult Transition ↔ Interpersonal Discomfort: 0.24 ***; Difficult Transition ↔ School Avoidance: 0.54 ***; and Interpersonal Discomfort ↔ School Avoidance: 0.23 **. The structural model includes a latent variable with a single item (the error variance of this item was set to zero).

The results for the direct effects are presented in Figure 1. These results showed that Anxious Anticipation was positively predicted by Educational Climate (β = 0.42, *p* ≤ 0.001) and negatively predicted by Sense of Belonging (β = −0.58, *p* ≤ 0.001). Difficult Transition was negatively predicted by Student–Teacher Relations (β = −0.16, *p* ≤ 0.05) and Sense of Belonging (β = −0.26, *p* ≤ 0.01). Interpersonal Discomfort was positively predicted by Educational Climate (β = 0.61, *p* ≤ 0.001) and negatively predicted by Sense of Belonging (β = −0.70, *p* ≤ 0.001). School Avoidance was positively predicted by Educational Climate (β = 0.29, *p* ≤ 0.05) and negatively predicted by Sense of Belonging (β = −0.28, *p* ≤ 0.05). School engagement was negatively predicted by Difficult Transition (β = −0.76, *p* ≤ 0.001). Underachievement was positively predicted by Difficult Transition (β = 0.26, *p* ≤ 0.05).

The present study shows that there was only one mediating effect. Specifically, an examination of the indirect effects, from school atmosphere to school engagement and poor performance, shows a significant indirect effect (Table 2), i.e., from a Sense of Belonging to school engagement via Difficult Transition (β = 0.20, *p* ≤ 0.05). The SEM analysis showed that the indicators were significant for each latent variable, with scores ranging from 0.61 to 0.95.

## 5. Discussion

Absenteeism is a major problem in the school context as it can become chronic to the point of becoming a real form of school refusal, which is closely associated with poor academic performance, delinquent behavior, and limited economic opportunities (Hendron & Kearney, 2016). Multiple family, emotional, and school-related factors underlie school refusal (Gallé-Tessonneau & Gana, 2019; Brouwer-Borghuis et al., 2019). Among school-related factors, school atmosphere may play a critical role in school attendance problems (Molinari & Grazia, 2023; Daily et al., 2020). However, there is little research on the relationship between the different dimensions of these constructs and school refusal in particular (Havik et al., 2015). Previous research has shown that perceiving an educational environment that fosters a Sense of Belonging and identification with the school promotes good interpersonal relationships with teachers and other students, where teachers care about them and support their development by providing a caring and well-structured learning environment with clear expectations; when students perceive that they are treated fairly by teachers, they are more engaged in learning and are more likely to attend school (Hamlin, 2021; Freeman et al., 2016). Conversely, students who perceive that the school environment does not meet their educational needs, that teachers exert excessive control and discipline over them, that the school atmosphere is characterized by high levels of distrust and disrespect between students and teachers, and that students do not care about each other, as well as those who feel that their presence in the classroom is not recognized or respected through equal opportunities, are more likely to experience feelings of inadequacy and anxiety about school, to avoid school, and to feel less engaged in school (Donat et al., 2018; Rizzotto & França, 2022; Glaesser et al., 2024), resulting in less satisfactory academic achievements or underachievement (Chere & Hlalele, 2014). Therefore, the vicious circle of absenteeism, where the pupil perceives the school atmosphere as negative and, therefore, tends to be absent more often, could lead to chronic forms of absenteeism (Gubbels et al., 2019), such as school refusal. However, no previous study has analyzed how the different components of school atmosphere contribute to school refusal and how these factors are related to school engagement and underachievement using a single model. Therefore, this study aimed to provide preliminary support for the indirect relationship between the five components of school atmosphere and school engagement and underachievement through school refusal. This study is also the first to examine the different components of school atmosphere simultaneously and separately, from relational as well as educational perspectives, and to relate them to the different forms of school refusal and how this may be related to school engagement and underachievement. Based on our hypothesis, we expected that school atmosphere (Student Relations, Student–Teacher Relations, Sense of Belonging, and Interpersonal Justice) could reduce school refusal and, consequently, promote school engagement and reduce underachievement. Furthermore, we hypothesized that Educational Climate could promote school refusal and, consequently, reduce school engagement and increase underachievement. The results partly confirmed our hypotheses. Based on the results obtained using SEM with latent variables, we found that students who perceive that the Educational Climate does not meet their educational needs and that teachers are overly competitive, controlling, and strict are more likely to experience anxiety about going to school and coping with the demands of school (Anxious Anticipation), to have difficulties with interpersonal relationships in the school context (Interpersonal Discomfort), and to have difficulty managing and regulating emotional and psychological distress expressed in avoidance and somatic symptoms (School Avoidance). These results confirm previous studies that have shown that the perception of a highly rigid and achievement-oriented Educational Climate with overly rigid teacher expectations and demands can promote typical symptoms of Anticipatory Anxiety in students (Zullig et al., 2011). Our results show how this type of Educational Climate can promote not only forms of school refusal related to Anticipatory Anxiety, as previous studies have shown, but also forms of school refusal related to difficulties in interpersonal relationships and the management and regulation of emotional and psychological distress at school. These findings can be explained if we consider that an Educational Climate based on rigid and controlling school discipline and a lack of appropriate or engaging teaching can lead pupils to perceive that the school environment does not meet their educational and autonomy needs (Van Eck et al., 2017; Karlberg et al., 2022), and this can lead them to experience anxiety in the face of school demands and manifest difficulties in managing and regulating their emotions, leading them to refuse to go to school. Our results also show that students who perceive a positive relationship with the teacher are less likely to experience difficulties in leaving their home and their parents to go to school (Difficult Transition). However, students who feel connected to school perceive that their needs for social connection and relationships with others are being met, and this may reduce the likelihood of developing forms of school refusal, ranging from Anxious Anticipation to Difficult Transition, Interpersonal Discomfort, and School Avoidance. These findings suggest that feeling connected to one’s school can help meet the developmental needs for social connection, relatedness, and belonging, which, in turn, can increase motivation to attend school and reduce absenteeism (Baskerville, 2020; Daily et al., 2020; Glaesser et al., 2024). The results show that a Sense of Belonging and teacher–student relationships are important factors in school refusal behavior due to difficulties in the transition from home to school. Consistent with the findings of Kim and Gentle-Genitty (2020), Gillen-O’Neel (2021), and Pope and Miles (2022), these results suggest that students have a greater ability to cope with the difficulties of transition and separation from home and their parents if they perceive a good relationship with teachers and a Sense of Belonging in the classroom. Our findings shed light on the relationship between the school atmosphere and various forms of school refusal, which has not been explored in depth in previous studies, and confirm previous results regarding the importance of school atmosphere in school attendance problems (Keppens & Spruyt, 2019; Grazia et al., 2024). Indeed, our results show that for forms of school refusal due to Transition Difficulties, Anxious Anticipation, Interpersonal Discomfort, and School Avoidance, a Sense of Belonging and positive relationships between students and teachers may act as mitigating factors, reducing the likelihood of these behaviors in adolescent students. Conversely, a rigid and highly controlling Educational Climate may promote school refusal due to Anxious Anticipation, Interpersonal Discomfort, and School Avoidance.

Previous research has shown how repeated absenteeism, especially prolonged and chronic absenteeism, can affect students’ academic performance and engagement at school (Benbenishty et al., 2016; Adams et al., 2016; Havik & Westergård, 2020). Indeed, frequent absenteeism can undermine student engagement in terms of time spent on homework and feelings, attachments, and connections to school, as well as self-regulation and learning strategies. Thus, prolonged absenteeism may reduce students’ engagement in school, but few studies have focused on understanding which forms of school refusal are more likely to reduce students’ engagement and increase underachievement. Our results show that school refusal due to difficulties in the transition from home to school (Difficult Transition) can reduce students’ engagement and lead to failures, suspensions, or underachievement. An indirect relationship underlies these findings. More specifically, our results confirm the role of Difficult Transition as a mediator between a Sense of Belonging and school engagement among adolescent students. These results suggest that if students feel that they belong and are connected to their school, probably because they perceive school as a place where they are welcome and which satisfies their need for connection and belonging, they are less likely to experience difficulties in the transition from home to school and difficulties in separating from their parents, which promotes students’ engagement in learning. Therefore, a Sense of Belonging to the class group can help students cope with transitional moments and promote student engagement (Kim & Gentle-Genitty, 2020; Pope & Miles, 2022). However, our results show that there is no relationship between two components of school atmosphere, namely, Interpersonal Justice and Student Relations, and school refusal. Previous research has shown that perceptions of a school where students are treated fairly and equitably by their teachers and the feeling that students can rely on each other are negatively correlated with absenteeism (Gubbels et al., 2019; Donat et al., 2018). Conversely, our study does not show a significant relationship between these two components of the school atmosphere and school refusal. It is likely that the fact that the different dimensions of school climate were disaggregated, considered in a single model, and related to the different forms of school refusal made it possible to identify the different relationships more specifically. Indeed, these results show that, among adolescents, the Educational Climate, Student–Teacher Relations, and Sense of Belonging are the main factors of the school atmosphere that are involved in school refusal. This study has some limitations that should be addressed in future studies. First, the cross-sectional nature of the design does not allow for causal associations. Therefore, future experimental and longitudinal studies may attempt to explore the causal direction of these associations. Furthermore, the convenience sample used in this study was recruited from a pool of high school students. Convenience sampling is a method of non-probability sampling that involves the participants being drawn from a close population group. The information collected from a convenience sample may not be representative of the way a generalized population group feels about any specific subject matter. Another limitation is the use of students’ self-reports. Individuals are often biased when they report on their own experiences. Future studies can include other methods of data collection in addition to self-reports, such as direct observations. Since school climate is a construct that is perceived by students, it can be examined through self-reporting. In addition, our results show the role of school refusal due to difficulties in the transition from home to school; so, it would be desirable for future studies to investigate the role of the family context (e.g., parental control and support, attachment styles, and parental self-efficacy) in promoting school refusal in adolescents. Despite these limitations, the present study makes an important contribution to the literature by expanding the knowledge on the factors that can influence school refusal and in the framework of the school atmosphere model. Our results also extend the knowledge of the factors that can influence students’ motivation to attend school, with important applications, especially to prevent negative repercussions on students’ functioning and well-being. This study also highlights the importance of structuring supportive and welcoming educational environments, where the training offered to students should aim to promote a Sense of Belonging in their school context and foster the perception of an educational context that meets their educational needs without resorting to excessive Educational Climate control. According to the literature, it is crucial to develop school support programs that actively involve both students and educational staff to promote student engagement and participation (Railsback, 2004; Finning et al., 2018). Indeed, educating students about school issues helps them to become more aware of the risks that may affect their education and promotes a greater understanding of the challenges they may face (Finning et al., 2018; Harris & Sass, 2011). In addition, it is essential to provide teachers with appropriate training and useful tools to reduce the pressure of school and to deal positively with the various stressful situations that students may experience (Finning et al., 2018; Kearney et al., 2023; Durham & Connolly, 2017). This approach should promote a view of learning as a process of growth and maturation. The training offered to teachers should aim to promote Student–Teacher Relations characterized by close relationships with their students and an Educational Climate conducive to student growth and self-development. Improving the school atmosphere can prevent students from developing forms of chronic absenteeism that could lead to their dropping out of school altogether and to future socioeconomic problems. This will facilitate the creation of a more positive school atmosphere, both from an educational and relational point of view, resulting in less school refusal behavior, greater school engagement, and more satisfactory achievement (Finning et al., 2018; Kearney et al., 2023). Therefore, offering this training can be useful in ensuring that adolescents perceive school as a comfortable, welcoming, and useful environment for their educational, emotional, and social growth; this also motivates them to attend school and feel involved in the learning process.

## 6. Conclusions

Our results distinguish between different components of the school atmosphere and school refusal and highlight how unsatisfactory academic achievements and engagement depend on how students perceive their educational environment. Therefore, improving students’ perceptions of the school atmosphere, promoting a sense of appearance and positive relationships with their teachers, and increasing the perception of an educational context that meets their educational needs without the need for excessive control or discipline may be an important intervention strategy to reduce school refusal and promote students’ engagement and school achievement. In conclusion, the results of our study confirm the important role of the school atmosphere in motivating students to attend school and engage in the learning process. Furthermore, our results underline that the mediating role of school refusal due to Transition Difficulties is important for understanding the relationship between Sense of Belonging and underachievement in school. Finally, these results show that different components of the school atmosphere may play different roles in school refusal and school achievement. However, this study only investigated the relationship between school atmosphere and school refusal; further studies are needed to improve the knowledge of this topic. In conclusion, these findings support our hypotheses by highlighting the multifaceted nature of the school atmosphere and its relationship to academic success. Furthermore, these findings highlight the complex interplay between emotional and academic factors involved in school absenteeism. By addressing specific components of the school atmosphere, such as fostering a Sense of Belonging, cultivating trusting relationships between teachers and students, and creating a supportive educational environment, schools can significantly reduce the risk of school refusal and promote academic success.

## Figures and Tables

**Table 1:** Correlation matrix showing Pearson correlation coefficients for the variables included in the study.

	1		2		3		4		5		6		7		8		9		10	
**1. Anxious Anticipation**	—																			
**2. Difficult Transition**	0.56	***	—																	
**3. Interpersonal Discomfort**	0.52	***	0.25	***	—															
**4. School Avoidance**	0.49	***	0.36	***	0.19	***	—													
**5. School Engagement**	−0.33	***	−0.54	***	−0.18	***	−0.16	***	—											
**6. Educational Climate**	0.19	***	0.20	***	0.19	***	0.13	**	−0.15	***	—									
**7. Student–Teacher Relations**	0.21	***	0.27	***	0.13	**	0.10	*	−0.20	***	0.57	***	—							
**8. Student Relations**	0.11	*	0.23	***	0.00		0.06		−0.18	***	0.56	***	0.54	***	—					
**9. Sense of Belonging**	0.27	***	0.29	***	0.24	***	0.12	**	−0.25	***	0.55	***	0.53	***	0.64	***	—			
**10. Interpersonal Justice**	0.16	***	0.22	***	0.04		0.11	*	−0.25	***	0.44	***	0.53	***	0.55	***	0.52	***	—	
**11. Underachievement**	0.17	***	0.14	**	0.01		0.39	***	−0.09	*	0.08		−0.03		0.02		0.03		−0.01	

Note. N = 528; * *p* < 0.05, ** *p* < 0.01, *** *p* < 0.001.

**Table 2:** Path estimates, SEs, and 95% CIs.

	β	SE	Lower Bound (BC) 95% CI	Upper Bound (BC) 95% CI	*p*
Direct Effect					
Educational Climate → Anxious Anticipation	0.42	0.07	0.12	0.42	≤0.001
Sense of Belonging → Anxious Anticipation	−0.58	0.06	−0.45	−0.21	≤0.001
Student–Teacher Relations → Difficult Transition	−0.16	0.07	0.00	−0.16	≤0.05
Sense of Belonging → Difficult Transition	−0.26	0.08	−0.04	−0.26	≤0.01
Educational Climate → Interpersonal Discomfort	0.61	0.06	−0.23	−0.69	≤0.001
Sense of Belonging → Interpersonal Discomfort	0.70	0.06	−0.23	−0.69	≤0.001
Educational Climate → School Avoidance	0.29	0.04	0.20	0.28	≤0.05
Sense of Belonging → School Avoidance	−0.28	0.04	−0.01	−0.27	≤0.05
Difficult Transition → School Engagement	−0.76	0.18	−0.81	−0.76	≤0.001
Difficult Transition → Underachievement	0.26	0.06	0.25	0.26	≤0.05
Indirect Effect via Difficult Transition					
Sense of Belonging → School Engagement	0.20	0.10	0.45	0.20	≤0.05

## Data Availability

The dataset analyzed during the current study is available from the corresponding author upon reasonable request.
